# The effect of febuxostat to prevent a further reduction in renal function of patients with hyperuricemia who have never had gout and are complicated by chronic kidney disease stage 3: study protocol for a multicenter randomized controlled study

**DOI:** 10.1186/1745-6215-15-26

**Published:** 2014-01-16

**Authors:** Tatsuo Hosoya, Kenjiro Kimura, Sadayoshi Itoh, Masaaki Inaba, Shunya Uchida, Yasuhiko Tomino, Hirofumi Makino, Seiichi Matsuo, Tetsuya Yamamoto, Iwao Ohno, Yugo Shibagaki, Satoshi Iimuro, Naohiko Imai, Masanari Kuwabara, Hiroshi Hayakawa

**Affiliations:** 1 Division of Nephrology and Hypertension, the Jikei University School of Medicine, 3-25-8, Nishishinbashi, Minato-ku, Tokyo 105-8461, Japan; 2 St. Marianna University School of Medicine, Kanagawa, Japan; 3 Tohoku University School of Medicine, Miyagi, Japan; 4 Osaka City University School of Medicine, Osaka, Japan; 5 Teikyo University School of Medicine, Tokyo, Japan; 6 Juntendo University School of Medicine, Tokyo, Japan; 7 Okayama University School of Medicine, Okayama, Japan; 8 Nagoya University School of Medicine, Aichi, Japan; 9 Hyogo college of Medicine, Hyogo, Japan; 10 The University of Tokyo Hospital, Clinical Research Support Center, Tokyo, Japan; 11 Toranomon Hospital, Tokyo, Japan

**Keywords:** Xanthine oxidase inhibitor, Urate-lowering therapy, Reduced renal function, Hyperuricemia, Chronic kidney disease, Randomized controlled study, Placebo

## Abstract

**Background:**

Hyperuricemia is a risk factor for the onset of chronic kidney disease (CKD) and is significantly associated with the progression of CKD. However, there is no sufficient evidence by interventional research supporting a cause-effect relationship. Hyperuricemic patients without gouty arthritis, whose serum urate (SUA) concentration is ≥8.0 mg/dL and who have a complication, are treated by pharmacotherapy in addition to lifestyle guidance. Nevertheless, there is no evidence that rationalizes pharmacotherapy for patients with hyperuricemia who have no complication and whose SUA concentration is below 9.0 mg/dL.

**Methods/Design:**

The FEATHER (*FE*buxostat *versus* placebo r*A*ndomized controlled *T*rial regarding reduced renal function in patients with *H*yperuricemia complicated by ch*R*onic kidney disease stage 3) study is a prospective, multicenter, double-blind, randomized, placebo-controlled trial of febuxostat—a novel, nonpurine, selective, xanthine oxidase inhibitor. The present study will enroll, at 64 medical institutions in Japan, 400 Japanese patients aged 20 years or older who have hyperuricemia without gouty arthritis, who present CKD stage 3, and whose SUA concentration is 7.1-10.0 mg/dL. Patients are randomly assigned to either the febuxostat or the control group, in which febuxostat tablets and placebo are administered orally, respectively. The dosage of the study drugs should be one 10-mg tablet/day at weeks 1 to 4 after study initiation, increased to one 20-mg tablet/day at weeks 5 to 8, and elevated to one 40-mg tablet/day at week 9 and then maintained until week 108. The primary endpoint is estimated glomerular filtration rate (eGFR) slope. The secondary endpoints include the amount and percent rate of change in eGFR from baseline to week 108, the amount and percent rate of change in SUA concentration from baseline to week 108, the proportion of patients who achieved an SUA concentration ≤6.0 mg/dL, and the incidence of renal function deterioration.

**Discussion:**

The present study aims to examine whether febuxostat prevents a further reduction in renal function as assessed with eGFR in subjects and will (1) provide evidence to indicate the inverse association between a reduction in SUA concentration and an improvement in renal function and (2) rationalize pharmacotherapy for subjects and clarify its clinical relevance.

**Trial registration:**

UMIN Identifier: UMIN000008343

## Background

Hyperuricemia is defined as 'serum urate (SUA) concentration >7.0 mg/dL’ [1]. The prevalence of hyperuricemia is 21.5% in Japanese adult men, and hyperuricemia prevails most in patients in their 30s and 40s; the prevalence of hyperuricemia is 30% in patients in their 30s [2]. Hyperuricemia is a risk factor for the onset of chronic kidney disease (CKD) [3] and is significantly associated with the progression of CKD [4,5]. Persisting hyperuricemia causes the tissue deposition of monosodium urate monohydrate crystals in extracellular fluids of the joints and other sites and induces urate deposition diseases (e.g., gouty arthritis, tophi, kidney injury, and urolithiasis) [6,7]. Women are at higher risk of experiencing the progression of reduced renal function at an SUA concentration ≥6.0 mg/dL that is lower than the conventional definition of hyperuricemia [3], and hyperuricemia influences the progression of kidney injury at the early phase of CKD [8]. In addition to the provision of lifestyle guidance (diet therapy, drinking restriction, encouragement of physical activity, patient education on treatment objective, and management of comorbidities), pharmacotherapy with urate-lowering drugs is recommended for (1) hyperuricemic patients with gouty arthritis or tophi, for (2) hyperuricemic patients whose SUA concentration is 8.0 mg/dL or higher and who have a complication (e.g., kidney injury, urolithiasis, hypertension, ischemic heart disease, diabetes mellitus, metabolic syndrome), and for (3) hyperuricemic patients whose SUA concentration is 9.0 mg/dL or higher and who have no complications [9].

Allopurinol, a purine inhibitor of xanthine oxidase (XO) conventionally used for urate-lowering therapy (ULT) to inhibit uric acid synthesis, was suggested to slow a reduction in renal function compared with the control group in a 12-month, prospective, randomized controlled trial (RCT) in 54 hyperuricemic patients with CKD [10] and delayed a reduction in renal function compared with the control group in a 24-month, prospective RCT in 113 patients with CKD stage 3 [11]. Because of the open-label nature, however, these small-size single-center clinical studies did not provide sufficient evidence about the effect of allopurinol to prevent renal function reduction.

A novel urate-lowering drug, febuxostat, is a potent non-purine selective inhibitor of XO and inhibits both the reduced and oxidized forms of the enzyme in contrast to allopurinol that inhibits the reduced form of the enzyme only [12]. Febuxostat is metabolized mainly by glucuronidation and oxidation in the liver [13], has its dual (urinary and fecal) pathways in excretion (urinary and fecal excretion rates: 49.1% and 44.9%, respectively) [14], and is effective and well tolerated in patients with mild to moderate renal and hepatic impairment [15]. A 28-day, Phase II, randomized clinical trial of febuxostat in 153 patients with gout [16] indicated that treatment with febuxostat significantly reduced SUA concentrations at doses of 40, 80, and 120 mg daily and was safe and well tolerated. The post-hoc analysis [17] of a clinical study on the long-term (5-year) oral administration of febuxostat in 116 patients with hyperuricemic gout [18] revealed that the maintenance of or an improvement in eGFR was inversely correlated with a quantitative reduction in SUA from baseline. Therefore, we presumed that continuous lowering of SUA concentrations might deter a further reduction in renal function of patients with hyperuricemia and/or gout and recognized the importance of prospectively evaluating the long-term effect of SUA reduction on renal function in hyperuricemic patients with impaired renal function.

The objective of the present study is to examine whether febuxostat prevents a further reduction in renal function as assessed with eGFR in Japanese patients with hyperuricemia, who have never had gout and are complicated by CKD stage 3.

## Methods/Design

### Study design and study organization

**Figure 1:**
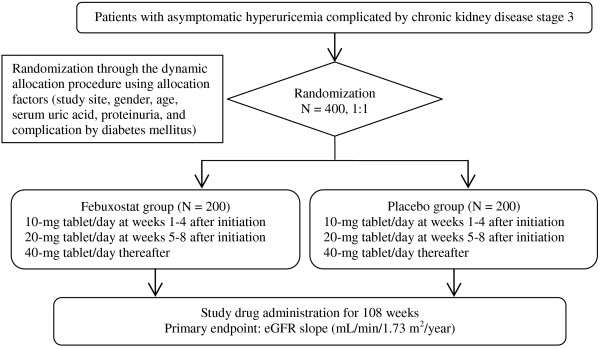
Flow diagram of the FEATHER study.

The FEATHER study is a prospective, multicenter, double-blind, randomized, placebo-controlled clinical study (Figure 1). The recruitment of patients started in November 2012 and concluded in December 2013. The scheduled study period including the 1-year recruitment period is 3 years. Steering Committee (Additional file 1) prepared the protocol and will assume the roles, including the solution of interpretational questions about the protocol, the adjustment of study details among medical institutions, and other tasks. Executive Committee will assume the roles, including the conduct and administration of the entire study, the regulatory check of study progress, the examination of study-propelling measures, the recognition of problems in conducting the study, the discussion on problem-solving measures, the adjustment of publication (article contribution and academic announcement), and other tasks. Independent Data and Safety Monitoring Committee will assume the roles, including the monitoring of study progress, the assessment of safety data at appropriate intervals, the recommendation to continue, adjust, or discontinue the study, and other tasks. The study was approved by Ethics Review Boards of the participating medical institutions (Additional file 2) and is being conducted according to the Declaration of Helsinki. All patients should provide written informed consent before enrollment.

### Patients

The FEATHER study will consecutively recruit, at 64 medical institutions in Japan, 400 Japanese patients with hyperuricemia who have never had gout and are complicated by CKD stage 3a or 3b, who are aged 20 years or older, and who have no gouty arthritis (Table 1).

**Table 1:** Inclusion and exclusion criteria

**Inclusion criteria**	**Exclusion criteria**
1. Men and women	1. Uncontrolled diabetes mellitus; HbA_1c_: ≥8.0% (JDS) or ≥8.4% (NGSP)
2. Age ≥20 years at informed consent	2. Systolic blood pressure ≥160 mmHg or diastolic blood pressure ≥100 mmHg
3. Patients with hyperuricemia; serum uric acid: 7.1-10.0 mg/dL	3. ALT or AST: greater than twice the upper limit of institutional reference range
4. eGFR: 30–59 mL/min/1.73 m^2^ (CKD stages 3a and 3b)	4. Change in serum creatinine level by more than 50% within 12 weeks before the confirmation of eligibility
5. No history of gout	5. Acute renal disease, nephrotic syndrome, other serious disease, dialysis, or renal transplantation
6. Written informed consent for study enrollment obtained from the patient	6. Complication or history of malignant tumor (not excluded from the study when the malignant tumor is not treated within 5 years and if there is no recurrence)
	7. History of hypersensitivity to febuxostat
	8. Intake of any one or more of the following drugs at the confirmation of eligibility: mercaptopurine hydrate, azathioprine, vidarabine, and didanosine
	9. Intake of any one or more of the following urate-lowering drugs within 4 weeks before the confirmation of eligibility: allopurinol, benzbromarone, probenecid, bucolome, and febuxostat
	10. Initiation of intake, dose modification, or discontinuation of intake of any one or more of the following drugs within 4 weeks before the confirmation of eligibility: losartan, fenofibrate, thiazide diuretics, and loop diuretics
	11. Continuous intake of salicylic acid drugs, e.g., aspirin (the patient taking low-dose aspirin [≤324 mg/day] need not to be excluded from the study)
	12. Hormone replacement therapy with estrogens
	13. Pregnancy, nursing, or planed pregnancy during the study
	14. Enrollment in other clinical trials within 24 weeks before providing informed consent
	15. Ineligibility at the investigator’s discretion

CKD, chronic kidney disease; Hba1c, hemoglobin A1c; JDS, Japan Diabetes Society; NGSP, National Glycohemoglobin Standardization Program.

### Treatment

#### Study treatment

Patients are randomly assigned to either of the febuxostat or the control group, in which febuxostat tablets and placebo are administered orally, respectively. Patients in the study groups will receive the allocated study drug for 108 weeks. The dose of the study drugs should be one 10-mg tablet/day at weeks 1 to 4 after study initiation, increased to one 20-mg tablet/day at weeks 5 to 8, and elevated to one 40-mg tablet/day at week 9 and then maintained until week 108.

#### Concomitant therapy

During the study period, the investigator provides the enrolled patient with lifestyle guidance (recommendation for diet management, restriction of alcohol consumption, and encouragement of exercise) by use of a leaflet prepared for this study. The administration of urate-lowering drugs (allopurinol, benzbromarone, probenecid, bucolome, and febuxostat except for study treatment) and contraindicated drugs of febuxostat (mercaptopurine hydrate, azathioprine, vidarabine, and didanosine) is prohibited during the study period.

#### Blinding

All parties involved in the present study (patients, investigators, and data analysts) will be blinded to the allocation of the study drugs—febuxostat tablets and placebo. Serum and urinary urate concentrations will be measured at the central laboratory. The measured values of serum and urinary urate will be concealed to the investigator and patient in an attempt to keep the allocation blinded. Laboratory tests at respective medical institutions will exclude the measurement of SUA concentration and urinary urate concentration from the assessment items. Furthermore, the following actions will be taken to ensure the safety of the patient when SUA concentrations change as described below:

1. The investigator will be informed when SUA concentrations measured at the central laboratory exceeded 12.0 mg/dL in a patient and will discontinue this study for the relevant patient;

2. Independent Data and Safety Monitoring Committee will monitor SUA concentrations and will examine a trend, if detected, to increase in SUA concentrations in each study group; and

3. The investigator will be informed when SUA concentrations decreased to 2.0 mg/dL or lower in a patient and will address the change appropriately.

#### Randomization

Patients, who meet all inclusion criteria and who do not fall under any exclusion criterion, are consecutively enrolled and randomly assigned at a 1:1 ratio to receive febuxostat or placebo through the dynamic allocation method using the following six background factors according to the minimization method: study site, gender, age (<65 or ≥65 years), SUA concentration (<8.0 mg/dL or ≥8.0 mg/dL), qualitative proteinuria (present or absent), and complication by diabetes (present or absent).

#### Endpoints

The primary endpoint of the FEATHER study is eGFR slope (change in eGFR per year, mL/min/1.73 m^2^/year) during the 108-week study period. The eGFR is calculated with the following equation defined by the Japanese Society of Nephrology: eGFRcreat (mL/min/1.73 m^2^) = 194 × [serum creatinine (mg/dLs) measured by the enzyme method]^-1.094^ × age^-0.287^ (× 0.739 if the patient is female).

The secondary endpoints are as follows: (1) the amount (mL/min/1.73 m^2^) and rate (%) of change in eGFR from baseline to weeks 24, 48, 72, and 108; (2) the amount (mg/dL) and the rate (%) of change in SUA concentration from baseline to week 108; (3) the proportion of patients whose SUA concentration became ≤6.0 mg/dL; (4) the incidence of renal deterioration defined by the commencement of dialysis or doubled serum creatinine concentration; (5) changes in biomarkers for renal function (serum cystatin C), oxidative stress (urinary 8-hydroxydeoxyguanosine and urinary L-type fatty acid-binding protein), and inflammation (serum C-reactive protein), as well as in variables for cardiovascular events (12-lead electrocardiogram, serum N-terminal pro-brain natriuretic protein, and albumin urine/creatinine ratio) to monitor the outcomes of ULT from baseline to week 108; (6) incidence of gouty arthritis; and (7) incidence of adverse events (AEs).

For safety assessment, AEs should be evaluated on clinical examination and in laboratory tests. The enrolled patient should visit the institution once every 4 weeks during the first 3 months and once every 3 months thereafter (Table 2). The investigator should evaluate patient compliance through history taking at each visit.

**Table 2:** Schedule for observation, testing, and assessment

		**Timing**													
**Items**		**Eligibility confirmation**	**Enrollment and assignment**	**Baseline**	**Treatment phase**										
Visit^a^		1		2	3	4	5	6	7	8	9	10	11	12	13
Week after treatment onset^a^		-4		0	4	8	12	24	36	48	60	72	84	96	108
Acquisition of informed consent		X													
Pregnancy test^b^, gender, and date of birth		X													
Smoking history, anamnesis, and complications				X											
Status of study drug intake					X	X	X	X	X	X	X	X	X	X	X
Status of concurrent therapy^c^		X		X	X	X	X	X	X	X	X	X	X	X	X
Commencement of dialysis					X	X	X	X	X	X	X	X	X	X	X
Development of gouty arthritis					X	X	X	X	X	X	X	X	X	X	X
Development of adverse events					X	X	X	X	X	X	X	X	X	X	X
Height (second visit only), body weight, and BMI				X						X					X
Blood pressure (systolic, diastolic) and pulse rate		X^d^		X	X	X	X	X	X	X	X	X	X	X	X
12-lead electrocardiogram				X^e^											X^f^
Hematology blood chemistry^g^, and urinalysis^h^		X^d^		X	X	X	X	X	X	X	X	X	X	X	X
Blood	Serum urate^i^ and creatinine concentrations	X^d^		√	√	√	√	√	√	√	√	√	√	√	√
	T-C, HDL-C, and TG			√ at fasting											
	HbA1c	X^j^		√						√					√
	Markers^k^			√						√					√
Urine	Urinary creatinine, albumin, and urate^i^ concentrations			√						√					√
	Markers^k^			√						√					√
Blood sampling volume for measurement at the central laboratory (mL)				16	10	10	10	10	10	16	10	10	10	10	16

X: Measurement at the institution’s laboratory; √: Measurement at the central laboratory.

^a^Corresponds to the time of completion, discontinuation, or withdrawal.

^b^For the premenopausal female patient only.

^c^Antihypertensives, antihyperlipidemics, antidiabetics, non-steroidal anti-inflammatory drugs, adrenocortical steroids, theophylline, colchicine, and part of contraindicated and restricted concurrent therapies.

^d^Blood pressure, serum urate concentration, serum creatinine concentration, alanine aminotransferase, and aspartate aminotransferase are required to confirm eligibility. Urine protein (qualitative) is required to adjust allocation.

^e^Data obtained within 6 months before consent acquisition should be acceptable, unless cardiovascular or other events have developed. Data within 3 months before and after study completion should be acceptable.

^f^Red blood cell count, white blood cell count, platelet count, hemoglobin, and hematocrit.

^g^Alanine aminotransferase, aspartate aminotransferase, γ-glutamyl transpeptidase, blood urea nitrogen, creatine kinase, and blood electrolytes (Na, K, and Cl).

^h^Urine protein, urine glucose, occult blood reaction, urine pH, and N-acetylglucosaminidase.

^i^Serum urate concentration and urinary urate concentration should be measured at the central laboratory only during the study period. Measurement results obtained at the central laboratory should be transmitted to the institutions, but serum urate concentration and urinary urate concentration should not.

^j^Should be measured for the patient who is suspected of having diabetes.

^k^Serum cystatin C, urinary 8-hydroxy-2’-deoxyguanosine, urinary liver-type fatty acid-binding protein, serum C-reactive protein, and serum N-terminal pro-brain-type natriuretic peptide.

BMI, body mass index; T-C, total cholesterol; HDLC, high-density lipoprotein cholesterol; TG, triglyceride.

### Statistical methods and sample size

The statistical analysis of the primary endpoint should be made to demonstrate the superiority of febuxostat to placebo. The linear regression line will be fitted to the longitudinal data of the eGFR for each patient. In patients with CKD stage 3, the intergroup difference in the eGFR slope should be compared between the febuxostat group and the control group according to Student’s t-test (analysis of variance), with the stratified adjustment of stages 3a and 3b. Subgroup analysis by stage 3a and 3b should be conducted to check the heterogeneity of the effect size.

Based on previous study results [11,19], we calculated a sample size of 89 patients to detect a difference of 2.7 mL/min/1.73 m^2^/year in eGFR slope between the study groups at each CKD stage (3a or 3b), with a standard deviation of 7.2, a power of 80%, and a one-sided significance level of 0.05. This sample size will provide 94.2% power at a two-sided significance level of 0.05 to perform intergroup comparisons in the overall cohort of patients with CKD stage 3 (178 patients per study group; 356 patients in total). Assuming a withdrawal rate of 11%, the target number of patients is 100 in the subgroups of patients with CKD 3a and 3b, respectively, leading to 200 patients required for the study groups, respectively, and to a total of 400 patients with CKD stage 3.

Efficacy analysis should be performed on an intention-to-treat principle basis. All patients, who took at least one dose of the study drug and who have at least one measurement of the eGFR after study drug administration, should be included in the efficacy analysis set. All patients who took at least one dose of the study drug should be included in the safety analysis set. The interim analysis is not planned in this study. The data to be collected in the present study will be managed and analyzed by the following organization that is completely independent from the present study’s organizations: Non-Profit Organization Japan Clinical Research Support Unit.

## Discussion

In addition to the traditional risk factors for cardiovascular disease (CVD) (e.g., smoking, older age, male gender, hypertension, diabetes mellitus, physical inactivity, menopause, and hyperlipidemia), reduced renal function is an important risk factor for CVD [20-25]. CKD, when advanced to stage 4 or 5, requires the commencement of dialysis and extensively elevates the risk of death from CVD. Recently, reduced renal function [26] and CKD stage 3 [27,28] were found to be risk factors for end-stage kidney disease (ESKD). In clinical settings, however, patients with CKD are more likely to die of CVD than to develop ESKD [29,30]. An eGFR below 60 mL/min/1.73 m^2^ is a risk factor for the onset of CVD [9], and reduced renal function increases the risk of developing cardiovascular death [31]. In Japan, nevertheless, pharmacotherapy is currently not recommended for hyperuricemic patients without gouty arthritis who have an SUA concentration below 8.0 mg/dL and for those without gouty arthritis who have no complication (e.g., CKD) and an SUA concentration below 9.0 mg/dL [2]. In Western countries, pharmacotherapy for asymptomatic hyperuricemia is not proactively recommended. This negative approach is attributable to the absence of evidence by interventional research on the causality between reduced renal function and the onset of CVD. The target SUA concentration is 6.0 mg/dL or below for hyperuricemic patients with gouty arthritis but is not established for patients with asymptomatic hyperuricemia. Therefore, hyperuricemic patients without gouty arthritis are managed fundamentally through lifestyle guidance to lower SUA concentrations in clinical settings. In patients with CKD stage 3, the causes of reduced renal function should be scrutinized, and multidisciplinary treatment is recommended to prevent renal function reduction [9].

Following the >40-year period during which allopurinol (marketed in 1966) was available as the only XO inhibitor, febuxostat was approved in 2009 in the USA for the chronic management of hyperuricemia in patients with gout. Oxypurinol, the active metabolite of allopurinol, exerts the XO-inhibitory activity (the Kd value: 0.54 nmol/L) by binding to the reduced form of XO (Mo(IV)) through a strong covalent bond [32]. However, the covalent bond disappears and oxypurinol is released because Mo (IV) is reoxidized with time and returns back to the oxidized form of XO (Mo (VI) whose half-life is 300 min at 25°C), and enzyme activity thus recovers [33]. Febuxostat presents striking contrast to oxypurinol because of its strong binding to enzyme proteins through multiple interactions, e.g., ionic bond, multiple hydrogen bonds, and hydrophobic interactions. Therefore, febuxostat does not depend on the oxidized or reduced form of XO and is strongly bound to both the oxidized and reduced forms of XO, thus inhibiting the enzyme for a long period of time and translating into its obvious therapeutic advantages [34]. Furthermore, febuxostat has high enzyme selectivity because of minimal effects on enzymes other than XO involved in the purine and pyrimidine metabolism [12,35]. Moreover, rat models, in which oxonic acid is used to induce hyperuricemia [36,37], indicate that hyperuricemia provokes a diversity of pathophysiological changes, e.g., activation of the renin-angiotensin system, decreased creatinine clearance, and severe arteriolopathy of the afferent arteriole [36-38]. Experimental studies afforded evidence that allopurinol and febuxostat, when used before the development of irreversible histological damage in the vasculature and glomeruli, can reverse these adverse changes, thereby preventing renal function reduction [39,40]. In addition, mild to moderate renal impairment has little effect on the pharmacodynamics and pharmacokinetics of febuxostat [41,42]. These experimental and clinical findings drove us to investigate the effect of early ULT with febuxostat on hyperuricemia complicated by renal impairment in clinical settings.

The present study, designed as a randomized placebo-controlled study and based on the presumed inverse correlation between eGFR and SUA concentration, is the first to prospectively assess the long-term (2-year) effect of SUA reduction on renal function in hyperuricemic patients with concurrent renal impairment. It is less common to prescribe allopurinol for gout patients with moderate renal impairment [43,44]. Hence, we consider that our study aiming at investigating the long-term effect of ULT with febuxostat to prevent a further reduction in renal function of hyperuricemic patients with moderately impaired renal function affords a new design approach and would be of clinical relevance.

Two large-scale RCTs published before the approval of febuxostat by the Food and Drug Administration [45,46] demonstrated that febuxostat 80 mg daily more effectively lowered SUA concentrations than did allopurinol 300 mg daily. In an 8-week, double-blind, randomized, allopurinol-controlled clinical trial in 244 patients with gout, febuxostat 40 mg daily showed a significantly more potent urate-lowering effect than allopurinol 200 mg daily [47]. A 6-month, large-scale, RCT of febuxostat 40/80 mg or allopurinol 300 mg (200 mg in moderate renal impairment) was conducted in 2,269 patients with gout and SUA ≥8.0 mg/dL [48]; the study indicated (1) the equivalent UL efficacy and comparable safety for febuxostat 40 mg daily and allopurinol 300/200 mg daily, (2) the significantly greater efficacy of febuxostat 40 mg daily in lowering SUA than allopurinol in patients with mildly or moderately impaired renal function, (3) comparable safety at the doses examined, and (4) the favorable tolerability of febuxostat 40 mg daily, especially for gout patients with mild or moderate renal impairment. The large-scale RCTs of febuxostat conducted to date [45,46,48] reported treatment-related AEs, the majority of which were mild to moderate in severity (e.g., liver function test abnormalities, diarrhea, nausea, headache, joint-related signs and symptoms, and rashes); the major serious AEs were non-specific bacterial infections, coronary artery disease, ischemic coronary artery disorders, and so on. Hence, there is a battery of experimental and clinical evidence to design an RCT in hyperuricemic patients with moderate renal impairment (30–59 mL/min/1.73 m^2^).

Our study has several limitations. Only hyperuricemic patients, who have never had gout and are complicated by CKD stage 3, are being enrolled in the present study. Therefore, no clinical evidence will be obtained for patients with severer CKD—stage 4 or 5. Under a beneficial medical insurance system in Japan (the universal healthcare insurance system), furthermore, patients who are willing to participate in a double-blind, randomized, placebo-controlled clinical study are represented by a particular population of patients with asymptomatic hyperuricemia complicated by stage 3 CKD. In this sense, selection bias cannot be ruled out.

In conclusion, we expect that the present study would show an inverse association between a reduction in SUA concentration and an improvement in renal function and will rationalize pharmacotherapy for subjects. Therefore, the early commencement of ULT for them may help to maintain renal function, to prevent the progression of CKD, and to improve renal prognosis in efforts to prevent CVD.

## Trial status

Recruitment and enrollment started in November 2012 and concluded in December 2013.

## Abbreviations

CKD: Chronic kidney disease; CVD: Cardiovascular disease; eGFR: Estimated glomerular filtration rate; FEATHER: *FE*buxostat versus placebo r*A*ndomized controlled *T*rial regarding reduced renal function in patients with *H*yperuricemia complicated by ch*R*onic kidney disease stage 3; SUA: Serum uric acid; ULT: Urate-lowering therapy; XO: Xanthine oxidase.

## Competing interests

The authors declare that they have no competing interests relevant to the manuscript beside the disclosures presented below.

## Authors’ contributions

TH and KK conceived the study design that was further developed by SI, MI, SU, YT, HM, SM, TY, IO, YS, SI, NI, NK, and HH. All authors read and approved the final version of the manuscript.

## Supplementary Material

Additional file 1 Lists of Steering Committee, Executive Committee, and Independent Data and Safety Monitoring Committee. Click here for file

Additional file 2 A list of 55 Ethics Review Boards that approved the FEATHER study. Click here for file
