# Supplementary material for: The effect of febuxostat to prevent a further reduction in renal function of patients with hyperuricemia who have never had gout and are complicated by chronic kidney disease stage 3: study protocol for a multicenter randomized controlled study
Source: Trials. 2014 Jan 16;15:26. doi: 10.1186/1745-6215-15-26 (PMC3899617; doi:10.1186/1745-6215-15-26)
Supplement: Additional file 1 — Lists of Steering Committee, Executive Committee, and Independent Data and Safety Monitoring Committee. [file 1745-6215-15-26-S1.docx]

Additional file 1 Lists of Steering Committee, Executive Committee, and Independent Data and Safety Monitoring Committee

Steering Committee: Tatsuo Hosoya (the Jikei University), Kenjiro Kimura

(St. Marianna University), Sadayoshi Itoh (Tohoku University), Masaaki Inaba

(Osaka City University), Yasuhiko Tomino (Juntendo University), Shin Fujimori (Teikyo University), Hirofumi Makino (Okayama University), Seiichi Matsuo

(Nagoya University), Hisashi Yamanaka (Institute of Rheumatology Tokyo Women’s Medical University), and Tetsuya Yamamoto (Hyogo College of Medicine)

Executive Committee: Iwao Ohno (the Jikei University), Yugo Shibagaki (St. Marianna University), Satoshi Iimuro (The University of Tokyo Hospital), Naohiko Imai (St. Marianna University), Masanari Kuwabara (Toranomon Hospital), and Hiroshi Hayakawa

(the Jikei University)

Independent Data and Safety Monitoring Committee: Tadao Akizawa

(Showa University), Tamio Teramoto (Teikyo University), Hiroshi Kasanuki

(Tokyo Women’s Medical University), and Kenichi Yoshimura (Kobe University)

Non-Profit Japan Clinical Research Support Unit: Presided by Yasuo Ohashi

(the University of Tokyo)
