# Supplementary material for: The effect of febuxostat to prevent a further reduction in renal function of patients with hyperuricemia who have never had gout and are complicated by chronic kidney disease stage 3: study protocol for a multicenter randomized controlled study
Source: Trials. 2014 Jan 16;15:26. doi: 10.1186/1745-6215-15-26 (PMC3899617; doi:10.1186/1745-6215-15-26)
Supplement: Additional file 2 — A list of 55 Ethics Review Boards that approved the FEATHER study. [file 1745-6215-15-26-S2.docx]

Additional file 2 A list of 55 Ethics Review Boards that approved the FEATHER Study

St. Marianna University School of Medicine; The Jikei University; Tohoku University Hospital;

Teikyo University; Juntendo University Hospital; Nagoya University Hospital;

Osaka City University; Hyogo College of Medicine; Okayama University; Tokushukai Group;

St. Luke’s International Hospital; Kyorin University; Fuji City General Hospital;

Osaki Citizen Hospital; Tokyo-kita Social Insurance Hospital; Nerima Hikarigaoka Hospital; Horinouchi Hospital; Ageo Central General Hospital; Tokyo Women’s Medical University;

Anjo Kosei Hospital; Yokkaichi Municipal Hospital; Jinshinkai Group, Shirasagi Hospital; Minami Osaka Hospital; Meijibashi Hospital; Hyogo Prefectural Amagasaki Hospital;

Chugoku Central Hospital; Kurashiki Central Hospital; Minami Tohoku Clinic;

Todachuo General Hospital; Nihon Koukan Hospital; Fujisawa City Hospital; Ehime Prefectural Central Hospital; Konan Kosei Hospital; Osaka University Hospital; Shimane University;

Fujita Health University; University of Tsukuba Hospital; Faculty of Medicine, Kagawa University; Yamanashi Prefectural Central Hospital; Japanese Red Cross Ashikaga Hospital;

Hiro Clinic Group; Kumamoto Social Insurance General Hospital; NTT Medical Center Tokyo;

Doujin Memorial Meiwa Hospital; National Hospital Organization Chiba-East-Hospital;

SATOU Hospital; Saitama Medical Center Jichi Medical University; Medical Toyou Ltd; National Disaster Medical Center; Kasugai Municipal Hospital; Yokohama City University Hospital; Shigei Medical Study Institute Hospital; Ryohoku Hospital; Showa University Northern Yokohama Hospital; Abe Clinic
